# Pleural Fluid Mesothelin as an Adjunct to the Diagnosis of Pleural Malignant Mesothelioma

**DOI:** 10.1155/2014/413946

**Published:** 2014-11-23

**Authors:** Jenette Creaney, Amanda Segal, Nola Olsen, Ian M. Dick, A. W(Bill) Musk, Steven J. Skates, Bruce W. Robinson

**Affiliations:** ^1^School of Medicine and Pharmacology, University of Western Australia, M503, 23 Stirling Highway, Crawley, Perth, WA 6009, Australia; ^2^National Centre for Asbestos Related Diseases, University of Western Australia, Nedlands, WA 6009, Australia; ^3^PathWest Laboratory Medicine, Queen Elizabeth II Medical Centre, Nedlands, WA 6009, Australia; ^4^Department of Respiratory Medicine, Sir Charles Gairdner Hospital, Nedlands, WA 6009, Australia; ^5^School of Population Health, University of Western Australia, Crawley, WA 6009, Australia; ^6^Biostatistics Center, Massachusetts General Hospital and Harvard Medical School, Boston, MA 02114, USA

## Abstract

*Rationale*. The diagnosis of pleural malignant mesothelioma (MM) by effusion cytology may be difficult and is currently controversial. Effusion mesothelin levels are increased in patients with MM but the clinical role of this test is uncertain. *Objectives*. To determine the clinical value of measuring mesothelin levels in pleural effusion supernatant to aid diagnosis of MM. *Methods and Measurements*. Pleural effusion samples were collected prospectively from 1331 consecutive patients. Mesothelin levels were determined by commercial ELISA in effusions and their relationship to concurrent pathology reporting and final clinical diagnosis was determined. *Results*. 2156 pleural effusion samples from 1331 individuals were analysed. The final clinical diagnosis was 183 MM, 436 non-MM malignancy, and 712 nonmalignant effusions. Effusion mesothelin had a sensitivity of 67% for MM at 95% specificity. Mesothelin was elevated in over 47% of MM cases in effusions obtained before definitive diagnosis of MM was established. In the setting of inconclusive effusion cytology, effusion mesothelin had a positive predictive value of 79% for MM and 94% for malignancy. *Conclusions*. A mesothelin-positive pleural effusion, irrespective of the identification of malignant cells, indicates the likely presence of malignancy and adds weight to the clinical rationale for further investigation to establish a malignant diagnosis.

## 1. Introduction

Pleural malignant mesothelioma (MM) is an aggressive asbestos-induced malignancy. The diagnosis of MM is often difficult [1] and may take several weeks to months to establish [2]. Current guidelines recommend that a diagnosis be based on demonstration of invasion by tumor cells [3]; however, not all patients are able to undergo invasive procedures to obtain a biopsy, and even when tissue is obtained diagnosis may still be difficult [4]. In MM, patients often present with a pleural effusion which can be used for diagnostic purposes. Although there is controversy regarding the cytological analysis of cells from pleural effusions, with sensitivities reported in several studies to be around 30%, the limitation of the technique relates primarily to difficulty in distinguishing MM from benign mesothelial cells [3, 5, 6]. However, a high sensitivity and specificity can be achieved if a standardized approach to cytodiagnosis is used [7].

In our institution, MM has been diagnosed by effusion cytology since 1974 [7–9] and a cytological diagnosis from an effusion sample in the appropriate clinical context has been accepted as the basis for patient management, for inclusion in clinical trials, and also for medicolegal purposes. Indeed in a recent review of these data, there were no false positive diagnoses of malignancy reported in the twenty year period reviewed where the gold standard was histology of biopsy or necropsy, together with clinical and radiological follow-up. Effusion cytology was performed in 63% of MM cases and demonstrated a positive predictive value of 99% for MM and 100% for malignancy [9].

Pleural effusions may arise in various clinical settings and identifying the cause of any effusion is important for determining subsequent treatment. The cause of an effusion can usually be established based upon clinical characteristics in association with imaging, biochemical, and microbiological analysis, together with cytological appearances. Although a diagnosis of cancer, that is, a malignant effusion, relies upon pathological analysis, in many patients the index of suspicion is not high enough to indicate the need for thoracoscopic or other intervention. Biomarkers produced by a suspected cancer which shed into the local effusion are a logical way to help guide the decision making of clinicians. This is because their local production means that levels are higher than in blood and thus they would be more likely to be an indication of the presence of cancer cells locally even if tumor cells are not identified by microscopic examination of the fluid. Also, biomarkers are easy and quick to measure. Previous studies have shown that soluble mesothelin levels in effusions have a diagnostic accuracy of over 70% [10–16] in MM. The clinical role for tumor biomarkers in pleural effusions has not been established so this study undertook to evaluate the clinical utility of measurement of mesothelin levels in pleural effusions which are not diagnostic of malignancy in a large prospectively collected, consecutive series. Analyses of these data indicate a clinical role for effusion mesothelin measurements in the diagnostic work-up of patients with pleural effusions.

## 2. Materials and Methods

### 2.1. Samples

Supernatants from pleural effusion samples, submitted to PathWest Laboratory Medicine (Queen Elizabeth II Medical Centre, Western Australia) between 2005 and 2010 for cytological analysis for malignancy were collected. Samples were centrifuged at 1000 g for 10 min at 4°C and supernatants stored at −80°C before analysis. Air dried and fixed smears and cell blocks were processed for standard diagnostic cytological assessment, and diagnoses were coded using the Systematized Nomenclature of Pathology (SNOP) system [17] by experienced cytopathologists [9]. In a subset of samples routine biochemical analyses were performed and nonmalignant effusions were classified as exudates or transudates on the basis of Light's criteria [18]. Effusions were classified as being associated with an infection if microorganisms were detected in the fluid or if the patient had concurrent pneumonia. Pathology reports, hospital records, and the records of the Western Australian MM Registry were retrospectively interrogated to establish a final diagnosis for each case. The MM registry evaluates all available data on an individual, including pathology, radiology, clinical findings, and asbestos exposure in making a final assessment as to whether a patient has MM [19]. Variables recorded for analysis included patient demographics, sample date, and SNOP code. SNOP codes were amalgamated into four categories: (i) normal, nondiagnostic, or nonmalignant; (ii) containing atypical cells or being suspicious of malignancy but not meeting the criteria of malignancy; (iii) malignant excluding MM; and (iv) MM. Date of diagnosis was recorded as the date of the first pathology report of malignancy, either based on effusion or biopsy specimen. This project was approved by the Sir Charles Gairdner Hospital Human Research Ethics Committee.

### 2.2. Quantification of Mesothelin

Soluble mesothelin concentrations were determined in duplicate following the manufacturer's instructions using the MESOMARK assay (Fujirebio Diagnostics Inc., Malvern, PA) and expressed in nanomolar (nM). All assays were performed on coded samples by investigators who were unaware of the patient's diagnosis, and mesothelin results were not reported to the clinical team at the request of the Ethics Committee. The limit of detection of the assay was 0.3 nM. A threshold value of 20 nM was used as the upper limit of normal as previously reported [12].

### 2.3. Statistical Analysis

Descriptive statistics and receiver operator characteristic (ROC) curves were performed using GraphPad Prism for Windows (GraphPad Software, San Diego, CA). Differences between groups of patients were assessed by Student's *t*-test after transforming mesothelin values to the log scale for which the distributions were closer to normality. For the same reason, median mesothelin values were estimated from the mean on the log scale and exponentiated to provide the estimate of the median on the original scale. Differences between pre- and postdiagnosis mesothelin levels were assessed by paired *t*-test. All reported *P* values are two sided. A level of *P* < 0.05 was accepted as significant.

## 3. Results

### 3.1. Subjects

Over the 67-month period of the study 2156 consecutive pleural effusion samples were collected from 1331 individuals, approximately 40% of whom were female. Data linkage combined with clinical follow-up revealed that there were 183 cases of confirmed MM. In over one third of MM cases (64/183 = 35%) the primary diagnosis was made on the cytological specimen with clinical and radiological data supporting the diagnosis. In 99 cases (i.e., 54%) a biopsy specimen confirmed MM diagnosis. Two cases were diagnosed at autopsy and the remaining 18 cases had diagnostic confirmation following electron microscopic review of the cytology sample. In summary there were 59 epithelioid, 23 biphasic, and 19 sarcomatoid MM plus a further 82 MM cases where histological subtype was not specified (Table 1).

There were 436 patients with effusions and non-MM malignancy, including 182 cases of primary lung cancer. There were 712 cases of effusions in patients without evidence of malignancy as determined from hospital records until death or for a median follow-up period of 11 months (range 0–90 months). Approximately 20% of these benign effusions were characterised as being transudates although all were submitted for cytopathological review. There was a higher ratio of males to females in the MM group relative to the other malignant and benign groups, consistent with the known gender imbalance in exposure to the carcinogen, asbestos. The distribution of patient ages was similar between the three groups (Table 1).

### 3.2. Pleural Effusion Soluble Mesothelin Concentrations Relative to Final Diagnosis

**Figure 1:**
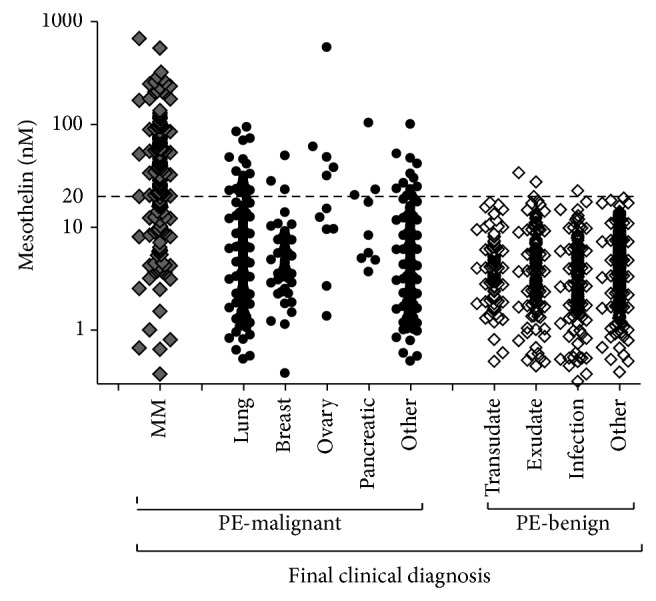
Soluble mesothelin concentrations in pleural effusion (PE) supernatants. Results presented for the single, most recent sample per individual against final clinical diagnosis (*n* = 1331). Dashed horizontal line indicates upper limit of normal threshold for effusion mesothelin (i.e., 20 nM).

**Figure 2:**
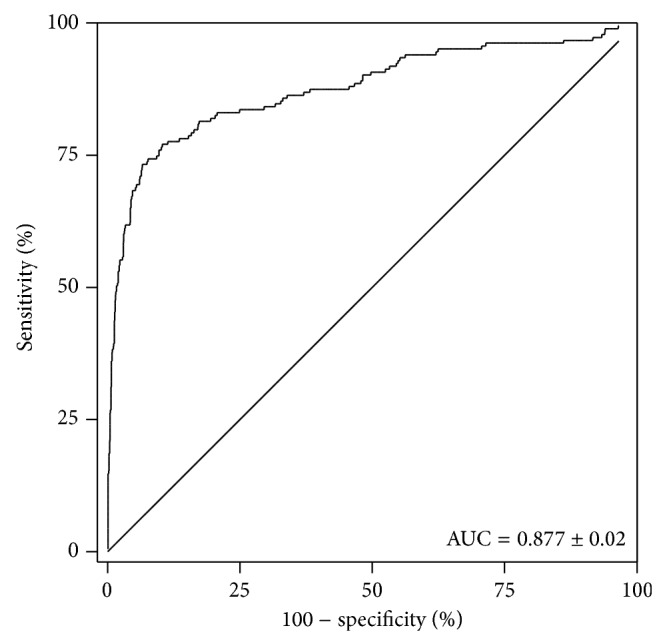
Receiver operating characteristic (ROC) curve showing accuracy of effusion mesothelin concentrations in the most recent sample per individual in differentiating all patients with MM (*n* = 183) from all other cases (*n* = 1148).

In order to determine sensitivity and specificity of effusion mesothelin for MM and limit bias in the results, analysis was performed on the most recent effusion sample received from each individual (*n* = 1331). Mesothelin concentrations in pleural effusions from patients with MM (28 ± 4.3 nM) were significantly higher than those from patients with benign effusions (3.2 ± 3 nM; *P* < 0.0001) and patients with non-MM malignancies (4.7 ± 3.9 nM; *P* < 0.0001) (Table 1; Figure 1). The diagnostic sensitivity and specificity of pleural effusion mesothelin for differentiating MM from all other causes of pleural effusion at a cut-off value of 20 nM was 67% (95% CI, 60 to 74%) and 95% (95% CI, 94 to 96%), respectively. Using ROC curve analysis, pleural effusion mesothelin generated an area under the curve (AUC) of 0.877 (95% CI, 0.843 to 0.91) for differentiating between patients with MM and all other patients in the study (Figure 2). Taken as a group, 11% of non-MM malignant effusions were mesothelin positive. Although absolute numbers were small, patients with primary ovarian or pancreatic cancers, two tumor types known to express mesothelin had relatively higher rates of mesothelin positivity than those with other malignancies.

There were 3 out of 712 (i.e., 0.4%) pleural effusions with mesothelin levels greater than 20 nM which were not associated with malignancy. Two of these three individuals were female and all were over 77 years of age (clinical characteristics are presented in Table 2). Two patients are deceased; one had a history of endometrial malignancy and no postmortem was performed to exclude the possibility of pleural malignancy; the second patient underwent necropsy examination and no pleural malignancy was identified. The third patient has a relatively short follow-up of 4 months.

### 3.3. Pleural Effusion Soluble Mesothelin Concentrations Relative to Cytology Report

**Figure 3:**
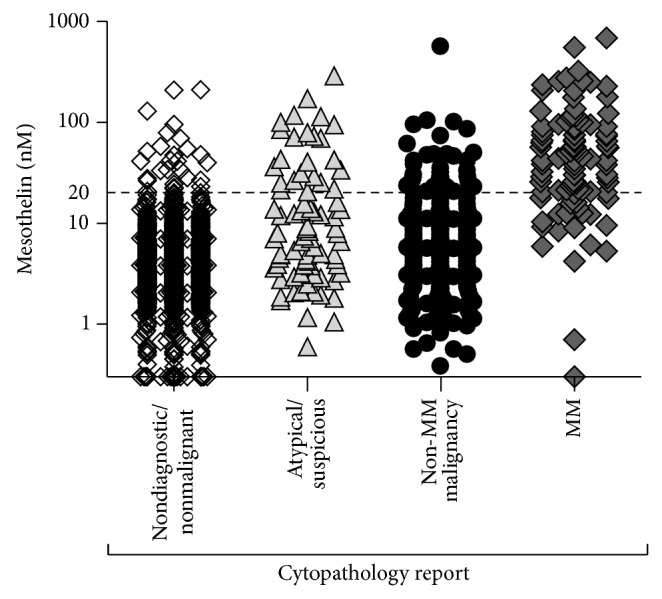
Pleural effusion soluble mesothelin concentrations: results for single, most recent sample per individual (*n* = 1331) plotted against the diagnosis reported by cytopathology for that sample.

In order to compare effusion mesothelin concentration with cytological diagnosis, pathology reports for the most recent sample per individual (*n* = 1331) were reviewed and related to effusion mesothelin levels. Approximately 64% of pleural effusions were classified as nonmalignant, 6% as atypical or suspicious of malignancy, 22% as non-MM malignancy, and 8% as MM. Mesothelin levels were elevated in 28 of 855 (i.e., 3%) samples reported as nonmalignant; 24 of 81 (i.e., 30%) atypical or suspicious samples; 42 of 291 (i.e., 14%) samples reported as non-MM malignancy; and 82 of 104 (i.e., 79%) samples reported as MM (Figure 3).

**Figure 4:**
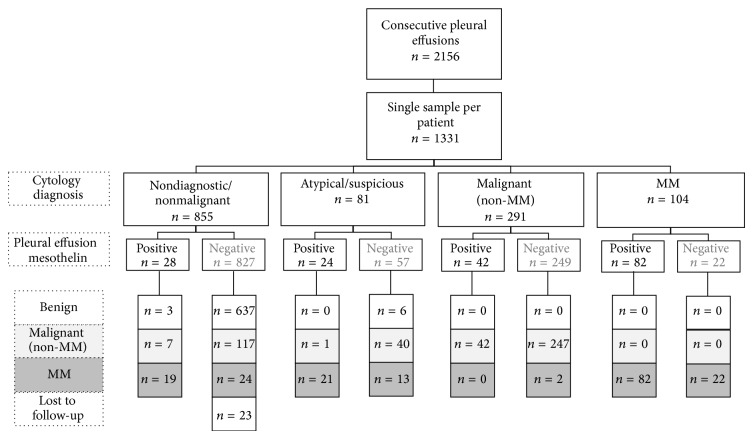
Flow diagram of the study. Number of cases for each category as defined by key (to the left of figure).

**Figure 5:**
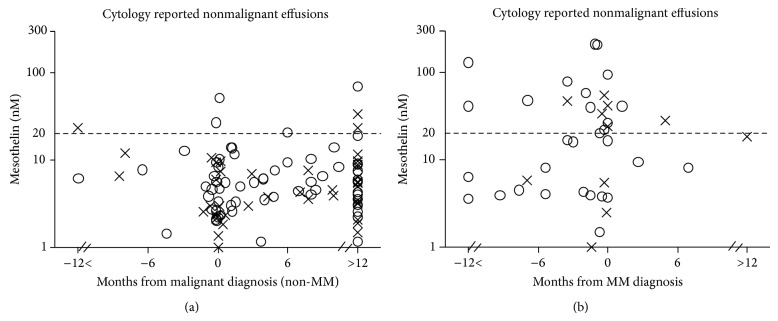
Soluble mesothelin concentrations in pleural effusion supernatants of patients with (a) a final clinical diagnosis of non-MM malignancy (*n* = 124) and (b) MM (*n* = 43), relative to the time of diagnosis. (×) Effusion samples with cytopathology report of normal or nondiagnostic; (⚪) effusion samples reported as being associated with inflammation or containing immune infiltrating cells.

Of the 855 pleural effusion samples which were classified as nonmalignant, follow-up was available on 832 (i.e., 97.3%). There were 167 samples in this group from patients with a known or subsequent diagnosis of malignancy, including 124 with non-MM malignancy and 43 with MM (Figure 4). For the non-MM cancer cases, approximately half of the effusions were called nondiagnostic because they consisted of blood only or were not of sufficient sample volume or quality for analysis, and the remaining half were noted to be associated with inflammation. Effusion mesothelin level was elevated in seven of these 124 patients including one patient who was diagnosed with metastatic pancreatic adenocarcinoma 14 months after the cytologically-negative/mesothelin-positive sample (Figure 5(a)).

There were 43 MM cases where the most recently received effusion sample was reported by the cytologist as being nondiagnostic or nonmalignant; the majority of these samples (70%) were noted to contain elevated numbers of inflammatory cells (Figure 5(b)). Most of these effusions were collected prior to diagnosis or concomitant with diagnosis made on a matching biopsy sample. Effusion mesothelin level was elevated in 19 of these patients including 17 out of 36 (i.e., 47%) before diagnosis made on later biopsy or cytology sample; two cases were elevated 16 months prior to diagnosis (Figure 5(b)).

**Figure 6:**
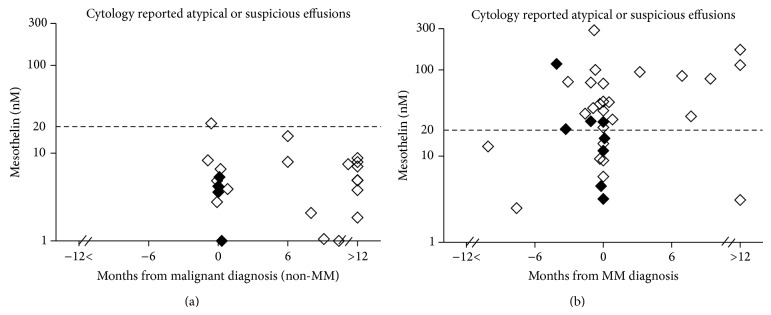
Soluble mesothelin concentrations in pleural effusion supernatants reported by cytopathology as being atypical (⋄) or suspicious of malignancy (◆) in patients with a final clinical diagnosis of (a) non-MM malignancy (*n* = 23) and (b) MM (*n* = 34), relative to the time of clinical diagnosis.

Of the 81 pleural effusion samples which were reported as being suspicious of malignancy or as containing atypical cells, 57 were from patients who had an existing diagnosis of malignancy. Clinical follow-up revealed that of the remaining 24 cases, 18 had died with a diagnosis of cancer 7 months (median and range; 0 to 57 months) after the sample was collected, and 6 remained alive 30 months (1 month to 8 years) after the sample was collected with no evidence of malignancy. Twenty-three effusion samples in this group were from patients with a recent or previous diagnosis of non-MM malignancy, including 8 with metastatic lung cancer and 4 with metastatic breast cancer. Effusion mesothelin level was only elevated in one of these samples and this was from a patient presenting with an effusion containing atypical cells associated with a primary lung adenocarcinoma (Figure 6(a)).

Thirty-four effusions from patients with a final diagnosis of MM were reported as atypical (*n* = 26) or suspicious of malignancy (*n* = 8), more than two-thirds of which were from before or near the time of diagnosis. Mesothelin level was elevated in the effusions of 15 of these 24 (62.5%) pre- or peridiagnosis samples (Figure 6(b)).

### 3.4. Positive Predictive Value of Mesothelin in Effusion Samples Not Definitely Diagnosed by Cytology as Malignant

Of the 1331 pleural effusion samples received from each individual, 936 were reported as being nondiagnostic, nonmalignant, atypical, or suspicious of malignancy (i.e., 855 + 81 = 936); mesothelin level was elevated in 53 (i.e., 5.7%) of these samples. Clinical follow-up revealed that 42 mesothelin positive samples were from MM patients, 8 mesothelin positive samples from patients with other malignancies, and 3 from people with no evidence of malignancy on follow-up. Therefore, the positive predictive value of effusion mesothelin level in effusion samples which were not definitively diagnosed by cytology was 79% for MM and 94% for any malignancy (Table 3).

### 3.5. Pleural Effusions Received from Patients with a Final Diagnosis of MM

**Figure 7:**
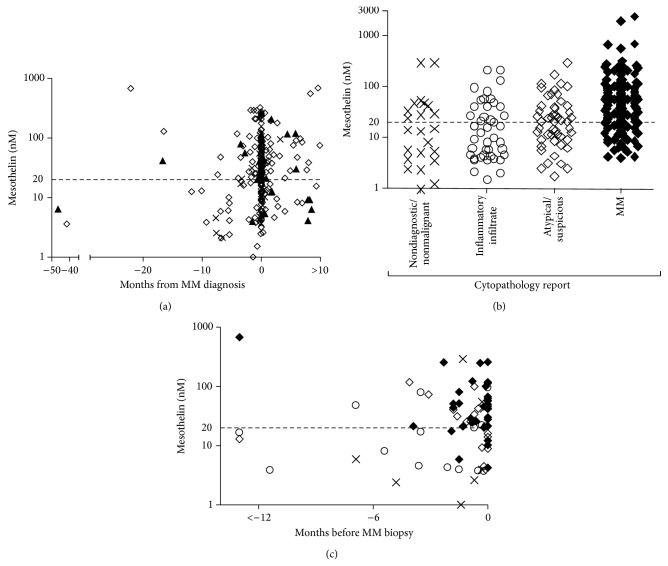
Pleural effusion soluble mesothelin concentrations in samples received from patients diagnosed with MM. (a) Mesothelin concentrations plotted relative to the time of diagnosis for MM patients with tumors of different histologies (×) sarcomatoid; (▲) biphasic; and (⚪) epithelial or nonspecified histology. Multiple samples per patient shown (*n* = 271). (b) Mesothelin concentrations plotted relative to the cytopathology report for each specimen. Multiple samples per patient shown (*n* = 271). (c) Mesothelin levels in effusions from 77 individuals with a biopsy confirmation of MM diagnosis. The pathology report for samples was (×) normal or nondiagnostic; (⚪) associated with inflammation or containing immune infiltrating cells; (⋄) atypical or suspicious of malignancy or (◆) MM.

A total of 271 pleural effusion samples from 183 individuals diagnosed with MM were collected. The majority of effusion specimens were within 1 month of the date of clinical diagnosis (mean ± SD; 1.1 ± 7.7 months). Effusion mesothelin levels were elevated in 170 of these 271 (63%) samples (Figure 7(a)), including in 8 of the 20 effusions from patients with sarcomatoid MM. Effusion mesothelin was elevated in 115 of the 174 effusions (66%) collected before or at the time of diagnosis. In three samples the mesothelin level was elevated over twelve months before diagnosis. Furthermore, mesothelin levels were elevated in 43% of samples that were not definitely diagnosed as MM by cytological assessment (Figure 7(b)). Nearly 80% (82/104) of effusions reported by cytological diagnosis as MM had elevated mesothelin levels (Figure 7(b)).

In a subset analysis of 77 cases where an effusion sample was available before or within one week of a biopsy confirming a diagnosis of MM (which included 7 sarcomatoid and 16 biphasic cases) pleural effusion mesothelin levels were elevated in 51 (66%) cases. In the 40 MM cases not reported as MM by cytology, mesothelin levels were elevated in 11/17 cases reported as atypical or suspicious; 6/15 of those reported as having an inflammatory infiltrate; and 3/8 nondiagnostic or negative samples (Figure 7(c)). As expected, the majority, that is, 60% of the 51 mesothelin-positive cases, were reported as MM following cytological examination.

## 4. Discussion

As the diagnosis of pleural MM can be difficult by effusion cytology or biopsy and may take weeks or months to establish, the use of effusion biomarkers has the potential to aid in diagnosis and thus to add to clinical decision making. In this retrospective study we show that elevated mesothelin level in an effusion is a strong predictive indicator of the presence of malignancy, particularly MM. Thus effusion mesothelin level may influence clinical decision making in patients with effusions.

Mesothelin levels have been examined in many series of serum samples [20]; however problems of confounding by kidney function [21, 22] and low sensitivity in presymptomatic individuals [23] have limited its acceptance as a diagnostic marker. In comparison there have been far fewer studies of the role of mesothelin measurements in pleural effusions in a diagnostic setting [10–16]. Tumor marker measurements in effusions have the potential to be more sensitive than serum measures because proximity of the fluid to the tumor allows continuous “sampling” of the tumor and because effusions are derived from an already symptomatic group.

The data from this study confirm and extend previous reports in a larger prospectively collected cohort [10, 12] that mesothelin levels greater than 20 nM in effusions are highly suggestive of malignancy, particularly of MM. With a sensitivity of 67% and specificity of 95% for distinguishing MM from all other effusions this study confirms that measurement of mesothelin level contributes to the diagnostic investigation of patients with pleural effusions. As 33% of MM cases do not have an elevated mesothelin level this test is not sufficient for diagnostic purposes as a stand-alone test. The finding that non-MM mesothelin positive cases are primarily due to other malignancies supports the notion that the value of this test is in differentiating malignant from nonmalignant effusions. We saw three “false positive” cases, that is, 0.4%; however only one of these cases underwent postmortem, and one has relatively short follow-up.

The incidence of MM in this study is relatively high because Western Australia has one of the highest per capita incidence rates of MM in the world, in part because of the extensive asbestos mining operations conducted in the town of Wittenoom in the north of the state [24, 25]. Furthermore, there is a relatively high percentage of MM cases where MM diagnosis is supported by assessment of cytological samples. In this setting nearly 80% of cytologically positive MM cases had an elevated effusion mesothelin level. However, the finding that mesothelin levels are elevated in 47% of MM cases in the absence of cytologically identifiable malignant cells and 62% of cases with atypical or suspicious cells, highlights the useful role that mesothelin can play in this setting. Indeed, in centres that lack experience in cytological diagnosis, the demonstration of elevated mesothelin levels in an effusion becomes even more important as an indicator of the likely presence of malignancy, particularly MM.

The clinical applicability of the test is most evident in situations where cytology is inconclusive or not routinely performed as 66% of biopsy-proven MM cases had elevated mesothelin levels in the effusions before diagnosis, with the test having a positive predictive value for MM of 79% and 94% for malignancy. Thus, the identification of an elevated mesothelin level in an effusion warrants further clinical investigation, which would most likely involve pleuroscopy with multiple biopsies. That is in areas of high MM prevalence for roughly every five operations performed four MM cases would be detected; the remaining case still likely to be malignant. In centres with lower rates of MM, pleuroscopy of patients with mesothelin positive effusions would be still worthwhile to identify malignancy of non-MM origin.

One consequence of combining measurement of effusion mesothelin level and cytopathology is earlier MM diagnosis. MM may present with nonspecific symptoms and there is sometimes a long interval of months to years [9] between presentation and subsequent diagnosis, which may in part be due to the presence of antecedent symptoms related to asbestos exposure. In different settings there are varying levels of suspicion for this cancer. Whilst the measurement of tumor markers in pleural effusions has not become routine clinical practice [26] there may be a clinical advantage in MM diagnosis in having a positive discriminatory test particularly in those centres not experienced in the diagnosis of this disease and where diagnosis is often complicated and time consuming. This approach may reduce hospital costs and the anxiety of waiting for a diagnosis. Further studies will be required to determine if such earlier diagnosis has any positive effect on treatment outcomes.

Limitations of this study include that MM cases were not all diagnosed based on review of biopsy specimens, indeed in many of the cases in which histological tissue was examined this was for treatment reasons (i.e., surgical staging) and not necessarily for diagnosis. A further confounding factor with this analysis was the presence of multiple samples per individual with different reported pathologies, relative to the time of diagnosis, rather than this being a case-control study. Although compounding the analysis this increases the applicability of the results to the true clinical situation.

As the clinical utility of the cytological diagnosis of MM has been called into question, the concomitant analysis of effusion tumour markers including mesothelin, CEA, Cyfra21-1, and others either alone or in combination may be a way to improve the clinical uptake of effusion-based MM diagnosis. This has the advantage of reducing the number of patients undergoing surgical procedures with the associated risks and costs.

**Figure 8:**
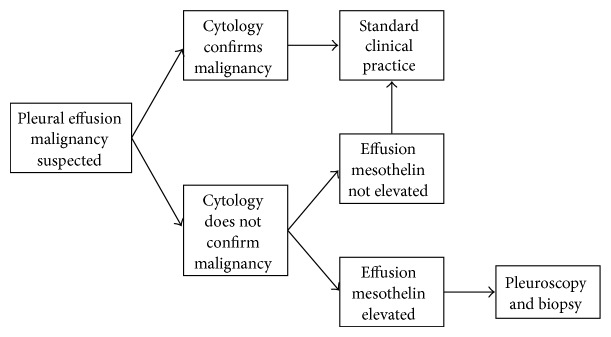
Suggested diagnostic flowchart. Suggested clinical role of effusion mesothelin estimation in patients with pleural effusions in whom malignancy, particularly mesothelioma, forms part of the differential diagnosis.

Given these data and acknowledging that the pretest probability of different malignancies varies between centres, we recommend the following general diagnostic approach to the analysis of effusion samples that are sent for cytological analysis (Figure 8). Firstly, if the cytological analysis provides sufficient information to establish the diagnosis of cancer, analysis of effusion mesothelin levels does not add anything. Secondly, if the cytological analysis is nondiagnostic or negative for malignancy, then effusion mesothelin level should be measured as the positive predictive value of the test for malignancy is high. What then happens will depend upon clinical circumstances, but if an elevated mesothelin level is detected the patient would typically undergo further imaging and thoracoscopic examination and biopsy. Thus, currently, patients in whom such an intervention might not otherwise be undertaken would undergo thoracoscopy. There are cases where a nondiagnostic effusion sample would not be followed up by a thoracoscopy. An elevated mesothelin level, when there is a low index of suspicion of malignancy, would increase the level of suspicion. Finally, if the cytological analysis shows atypical or suspicious cells, measurement of pleural fluid mesothelin might be sufficient to support the cytological diagnosis and will expedite further clinical decision making, especially regarding thoracoscopy and biopsy.

## Figures and Tables

**Table 1:** Final diagnosis, patient characteristics, and mesothelin results.

Final diagnosis	*n*	Female, *n* (%)	Age, mean (range)	Mesothelin^1,2^ nM	Mesothelin positive^3^ *n* (%)
MM	**183**	**26 (14%)**	**71 (45–97)**	**28 ± 4.3**	**122 (67%)**
Non-MM malignancy all	**436**	**226 (52%)**	**70 (24–97)**	**4.7 ± 3.9** ^***^	**50 (11%)**
Lung cancer	182	78 (43%)	72 (36–97)	5.2 ± 3.6	26 (14%)
Breast cancer	62	61 (98%)	66 (37–96)	4.1 ± 3.3	3 (5%)
Ovarian cancer	11	11 (100%)	64 (42–77)	19 ± 5.1	5 (45%)
Pancreatic cancer	10	2 (20%)	68 (56–77)	6.3 ± 8.4	3 (30%)
Other malignancies	171	74 (44%)	68 (24–94)	3.9 ± 4	13 (8%)
Nonmalignant all	**712**	**260 (37%)**	**69 (18–99)**	**3.2 ± 3** ^***^	**3 (0.4%)**
Transudate	141	58 (41%)	74 (34–99)	3.4 ± 2.7	0 (0%)
Exudate	129	44 (34%)	67 (19–94)	3.4 ± 3.5	2 (2%)
Infection	165	58 (35%)	65 (29–96)	2.3 ± 3.8	1 (0.6%)
Benign—not specified	277	106 (38%)	70 (18–96)	3.5 ± 3.1	0 (0%)

^1^expontiated mean of log transformed data plus/minus standard error of log transformed data ∗ 100, in the most recent sample received per patient.

^2^significant difference between indicated cohorts and the mesothelioma cohort as a whole (*n* = 183) as determined by Student's *t*-test (^***^is *P* < 0.0001).

^3^Number of individuals in whom soluble mesothelin was >20 nM in the most recent sample received per patient.

**Table 2:** Characteristics of patients with positive effusion mesothelin and no evidence of malignancy.

Mesothelin (nM)	Age	Sex	Condition associated with effusion and comorbidities	Status	Time to follow-up	Asbestos exposed	Autopsy result
23	85	F	Tuberculosis	Deceased	4 days	Yes	ND

34	89	F	Idiopathic (chronic bronchitis) Previous history of endometrial cancer (10 years ago)	Alive	4 months	No	

28	77	M	Bilateral bronchopneumonia pleural plaques	Deceased	13 months	Yes	No malignancy

**Table 3:** Mesothelin concentrations in effusions nondiagnostic for malignancy by cytology relative to final clinical diagnosis.

Mesothelin	Condition				Total
	MM	Non-MM malignancy	Nonmalignant	Inadequate follow-up	
Positive	42	8	3	0	53
Negative	35	139	686	23	883
Total	**77**	**147**	**689**	**23**	**936**

PPV_MM_ = 42/53 = 79%.

PPV_malignancy_ = (42 + 8)/53 = 94%.

## References

[1] Addis B., Roche H. (2009). Problems in mesothelioma diagnosis. *Histopathology*.

[2] Chahinian A. P., Pajak T. F., Holland J. F., Norton L., Ambinder R. M., Mandel E. M. (1982). Diffuse malignant mesothelioma. Prospective evaluation of 69 patients. *Annals of Internal Medicine*.

[3] Husain A. N., Colby T. V., Ordonez N. G., Krausz T., Borczuk A., Cagle P. T., Chirieac L. R., Churg A., Galateau-Salle F., Gibbs A. R., Gown A. M., Hammar S. P., Litzky L. A., Roggli V. L., Travis W. D., Wick M. R. (2009). Guidelines for pathologic diagnosis of malignant mesothelioma: a consensus statement from the International Mesothelioma Interest Group. *Archives of Pathology and Laboratory Medicine*.

[4] Ordóñez N. G. (2007). What are the current best immunohistochemical markers for the diagnosis of epithelioid mesothelioma? A review and update. *Human Pathology*.

[5] Renshaw A. A., Dean B. R., Antman K. H., Sugarbaker D. J., Cibas E. S. (1997). The role of cytologic evaluation of pleural fluid in the diagnosis of malignant mesothelioma. *Chest*.

[6] Stahel R. A., Weder W., Felip E. (2008). Malignant pleural mesothelioma: ESMO clinical recommendations for diagnosis, treatment and follow-up. *Annals of Oncology*.

[7] Whitaker D. (2000). The cytology of malignant mesothelioma. *Cytopathology*.

[8] Wolanski K. D., Whitaker D., Shilkin K. B., Henderson D. W. (1998). The use of epithelial membrane antigen and silver-stained nucleolar organizer regions testing in the differential diagnosis of mesothelioma from benign reactive mesothelioses. *Cancer*.

[9] Segal A., Sterrett G. F., Frost F. A., Shilkin K. B., Olsen N. J., Musk A. W., Nowak A. K., Robinson B. W. S., Creaney J. (2013). A diagnosis of malignant pleural mesothelioma can be made by effusion cytology: results of a 20 year audit. *Pathology*.

[10] Davies H. E., Sadler R. S., Bielsa S., Maskell N. A., Rahman N. M., Davies R. J. O., Ferry B. L., Lee Y. C. G. (2009). Clinical impact and reliability of pleural fluid mesothelin in undiagnosed pleural effusions. *American Journal of Respiratory and Critical Care Medicine*.

[11] Pass H. I., Wali A., Tang N. (2008). Soluble mesothelin-related peptide level elevation in mesothelioma serum and pleural effusions. *Annals of Thoracic Surgery*.

[12] Creaney J., Yeoman D., Naumoff L. K., Hof M., Segal A., Musk A. W., De Klerk N., Horick N., Skates S. J., Robinson B. W. S. (2007). Soluble mesothelin in effusions: a useful tool for the diagnosis of malignant mesothelioma. *Thorax*.

[13] Scherpereel A., Grigoriu B., Conti M., Gey T., Grégoire M., Copin M.-C., Devos P., Chahine B., Porte H., Lassalle P. (2006). Soluble mesothelin-related peptides in the diagnosis of malignant pleural mesothelioma. *The American Journal of Respiratory and Critical Care Medicine*.

[14] Grigoriu B., Chahine B., Zerimech F., Grégoire M., Balduyck M., Copin M.-C., Devos P., Lassalle P., Scherpereel A. (2009). Serum mesothelin has a higher diagnostic utility than hyaluronic acid in malignant mesothelioma. *Clinical Biochemistry*.

[15] Alemán C., Porcel J. M., Segura R. M., Alegre J., Esquerda A., Ruiz E., Bielsa S., de Sevilla T. F. (2009). Pleural fluid mesothelin for the differential diagnosis of exudative pleural effusions. *Medicina Clinica*.

[16] Yamada S., Tabata C., Tabata R., Fukuoka K., Nakano T. (2011). Clinical significance of pleural effusion mesothelin in malignant pleural mesothelioma. *Clinical Chemistry and Laboratory Medicine*.

[17] College of American Pathologists Committee on Nomenclature and Classification of Disease (1965). *Systematized Nomenclature of Pathology*.

[18] Light R. W., Macgregor M. I., Luchsinger P. C., Ball W. C. (1972). Pleural effusions: the diagnostic separation of transudates and exudates. *Annals of Internal Medicine*.

[19] Threlfall T., Thompson J., Olsen N. (2005). *Cancer in Western Australia: Incidence and Mortality 2003 and Mesothelioma 1960–2003*.

[20] Tung A., Bilaceroglu S., Porcel J., Creaney J., Lee Y. (2011). Biomarkers in pleural diseases. *US Respiratory Disease*.

[21] Boudville N., Paul R., Robinson B. W. S., Creaney J. (2011). Mesothelin and kidney function—analysis of relationship and implications for mesothelioma screening. *Lung Cancer*.

[22] Hollevoet K., Bernard D., de Geeter F. (2009). Glomerular filtration rate is a confounder for the measurement of soluble mesothelin in serum. *Clinical Chemistry*.

[23] Creaney J., Olsen N. J., Brims F., Dick I. M., Musk A. W., De Klerk N. H., Skates S. J., Robinson B. W. S. (2010). Serum mesothelin for early detection of asbestos-induced cancer malignant mesothelioma. *Cancer Epidemiology Biomarkers and Prevention*.

[24] Leigh J., Driscoll T. (2003). Malignant mesothelioma in Australia, 1945–2002. *International Journal of Occupational and Environmental Health*.

[25] Musk A. W., de Klerk N. H. (2004). Epidemiology of malignant mesothelioma in Australia. *Lung Cancer*.

[26] Light R. W. (2004). Tumor markers in undiagnosed pleural effusions. *Chest*.

